# HPLC-MS/MS Analyses Show That the Near-Starchless *aps1* and *pgm* Leaves Accumulate Wild Type Levels of ADPglucose: Further Evidence for the Occurrence of Important ADPglucose Biosynthetic Pathway(s) Alternative to the pPGI-pPGM-AGP Pathway

**DOI:** 10.1371/journal.pone.0104997

**Published:** 2014-08-18

**Authors:** Abdellatif Bahaji, Edurne Baroja-Fernández, Ángela María Sánchez-López, Francisco José Muñoz, Jun Li, Goizeder Almagro, Manuel Montero, Pablo Pujol, Regina Galarza, Kentaro Kaneko, Kazusato Oikawa, Kaede Wada, Toshiaki Mitsui, Javier Pozueta-Romero

**Affiliations:** 1 Instituto de Agrobiotecnología, Universidad Pública de Navarra/Consejo Superior de Investigaciones Científicas/Gobierno de Navarra, Mutiloabeti, Nafarroa, Spain; 2 Servicio de Apoyo a la Investigación, Universidad Pública de Navarra, Campus de Arrosadia, Iruña, Nafarroa, Spain; 3 Department of Applied Biological Chemistry, Niigata University, Niigata, Japan; Leibniz-Institute for Vegetable and Ornamental Plants, Germany

## Abstract

In leaves, it is widely assumed that starch is the end-product of a metabolic pathway exclusively taking place in the chloroplast that (a) involves plastidic phosphoglucomutase (pPGM), ADPglucose (ADPG) pyrophosphorylase (AGP) and starch synthase (SS), and (b) is linked to the Calvin-Benson cycle by means of the plastidic phosphoglucose isomerase (pPGI). This view also implies that AGP is the sole enzyme producing the starch precursor molecule, ADPG. However, mounting evidence has been compiled pointing to the occurrence of important sources, other than the pPGI-pPGM-AGP pathway, of ADPG. To further explore this possibility, in this work two independent laboratories have carried out HPLC-MS/MS analyses of ADPG content in leaves of the near-starchless *pgm* and *aps1* mutants impaired in pPGM and AGP, respectively, and in leaves of double *aps1/pgm* mutants grown under two different culture conditions. We also measured the ADPG content in wild type (WT) and *aps1* leaves expressing in the plastid two different ADPG cleaving enzymes, and in *aps1* leaves expressing in the plastid GlgC, a bacterial AGP. Furthermore, we measured the ADPG content in *ss3/ss4/aps1* mutants impaired in starch granule initiation and chloroplastic ADPG synthesis. We found that, irrespective of their starch contents, *pgm* and *aps1* leaves, WT and *aps1* leaves expressing in the plastid ADPG cleaving enzymes, and *aps1* leaves expressing in the plastid GlgC accumulate WT ADPG content. In clear contrast, *ss3/ss4/aps1* leaves accumulated ca. 300 fold-more ADPG than WT leaves. The overall data showed that, in Arabidopsis leaves, (a) there are important ADPG biosynthetic pathways, other than the pPGI-pPGM-AGP pathway, (b) pPGM and AGP are not major determinants of intracellular ADPG content, and (c) the contribution of the chloroplastic ADPG pool to the total ADPG pool is low.

## Introduction

Starch is a branched homopolysaccharide of α-1,4-linked glucose subunits with α-1,6-linked glucose at the branched points. Synthesized by starch synthases (SSs) using ADPglucose (ADPG) as the sugar donor molecule, this polyglucan accumulates as predominant storage carbohydrate in most plants. In leaves, up to 50% of the photosynthetically fixed carbon is retained within the chloroplasts of mesophyll cells during the day to synthesize starch [1], [2], which is then remobilized during the subsequent night to support non-photosynthetic metabolism and growth by continued export of carbon to the rest of the plant. Due to the diurnal rise and fall cycle of its levels, foliar starch is termed “transitory starch”.

**Figure 1:**
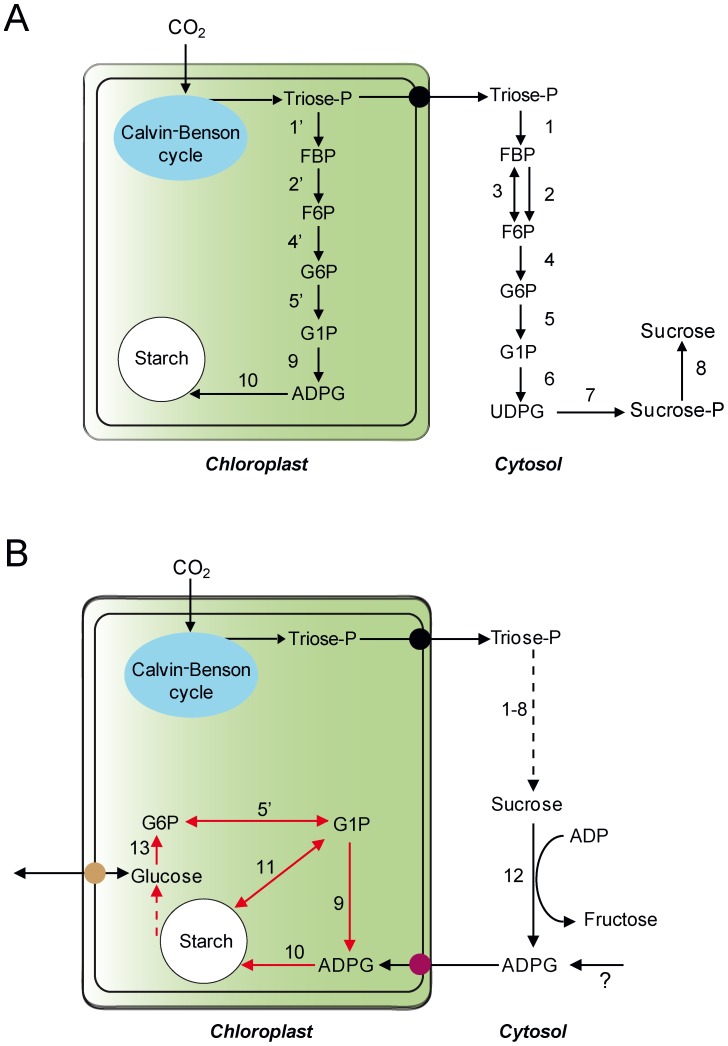
Suggested models of starch biosynthesis in leaves. (A) The classic model of starch biosynthesis according to which (a) the starch biosynthetic process takes place exclusively in the chloroplast, segregated from the sucrose biosynthetic process taking place in the cytosol, and (b) AGP exclusively catalyzes the synthesis of ADPG. (B) Suggested “additional/alternative” model of starch biosynthesis wherein (a) ADPG is produced in the cytosol by enzyme(s) such as SuSy and then is transported to the chloroplast by the action of an ADPG translocator, and (b) pPGM and AGP play an important role in the scavenging of glucose units derived from starch breakdown. Starch to glucose conversion would involve the coordinated actions of amylases, isoamylase and disproportionating enzyme [21]–[23]. According to this interpretation of transitory starch biosynthesis starch accumulation in leaves is the result of the balance between *de novo* starch synthesis from ADPG entering the chloroplast and breakdown, and the efficiency by which starch breakdown products are recycled back to starch by means of pPGM and AGP. Thus, this view predicts that the recovery towards starch biosynthesis of the glucose units derived from the starch breakdown will be deficient in pPGM and AGP mutants, resulting in a parallel decline of starch accumulation and enhancement of soluble sugars content since starch breakdown derived products (especially glucose) will leak out the chloroplast through the very active glucose translocator [24]. The enzyme activities involved are numbered as follows: 1, 1′, fructose-1,6-bisphosphate aldolase; 2, 2′, fructose 1,6-bisphosphatase; 3, PPi:fructose-6-phosphate phosphotransferase; 4, 4′, PGI; 5, 5′, PGM; 6, UDPG pyrophosphorylase; 7, sucrose phosphate synthase; 8, sucrose-phosphate-phosphatase; 9, AGP; 10, SS; 11, starch phosphorylase; 12, SuSy; 13, plastidial hexokinase [25], [26]. FBP: fructose bis-phosphate; UDPG: UDP-glucose.

It is widely assumed that the whole starch biosynthetic process occurring in mesophyll cells of leaves resides exclusively in the chloroplast [3]–[5]. According to this classical view of starch biosynthesis, starch is considered the end-product of a metabolic pathway that is linked to the Calvin-Benson cycle by means of the plastidic phosphoglucose isomerase (pPGI). This enzyme catalyzes the conversion of fructose-6-phosphate from the Calvin-Benson cycle into glucose-6-phosphate (G6P), which is then converted into glucose-1-phosphate (G1P) by the plastidic phosphoglucomutase (pPGM). ADPG pyrophosphorylase (AGP) then converts G1P and ATP into inorganic pyrophosphate and ADPG necessary for starch biosynthesis (**Figure 1A**). These three enzymatic steps are reversible, but the last step is rendered irreversible upon hydrolytic breakdown of PPi by plastidial alkaline pyrophosphatase.

The classic view of transitory starch biosynthesis also implies that AGP is the sole source of ADPG, and functions as the major regulatory step in the starch biosynthetic process [3]–[7]. Plant AGPs are heterotetrameric enzymes comprising two types of homologous but distinct subunits, the small (APS) and the large (APL) subunits [9], [10]. In Arabidopsis, six genes encode proteins with homology to AGP. Two of these genes (*APS1* and *APS2*) code for small subunits, and four (*APL1-APL4*) encode large subunits [9]–[11]. *APS2* is in a process of pseudogenization [12] since its expression level is two orders of magnitude lower than that of *APS1* [10], and its product lacks activity due to the absence of essential amino acids involved in the catalysis and/or in the binding of G1P and 3-phosphoglycerate [9]. Whereas APS1, APL1 and APL2 are catalytically active, APL3 and APL4 have lost their catalytic properties during evolution [13]. In Arabidopsis, the large subunits are highly unstable in the absence of small subunits [14]. Therefore, *APS1* null mutants lack not only APS1, but also the large subunits, which results in a total lack of AGP activity [13], [15].

In *Arabidopsis,* genetic evidence showing that transitory starch biosynthesis occurs solely by the pPGI-pPGM-AGP pathway has been obtained from the characterization of mutants impaired in pPGI [16], [17], pPGM [18], [19] and AGP [13], [14], [20]. Despite the monumental amount of data apparently supporting the classic interpretation of transitory starch biosynthesis in mesophyll cells involving the pPGI-pPGM-AGP pathway (**Figure 1A**), mounting evidence has exposed inconsistencies that previews the occurrence of important pathway(s) of transitory starch biosynthesis wherein (a) pPGI plays a minor role in the connection of the Calvin-Benson cycle with the starch biosynthetic pathway, (b) a sizable pool of ADPG linked to starch biosynthesis is produced in the cytosol by enzymes such as sucrose synthase (SuSy) [27]–[32], (c) cytosolic ADPG is transported to the chloroplast by the action of a yet to be identified ADPG translocator [33], and (d) pPGM and AGP play important roles in the scavenging of glucose units derived from starch breakdown occurring during starch biosynthesis and during the biogenesis of the starch granule [27], [28], [32], [34]. According to this interpretation of transitory starch biosynthesis (schematically illustrated in **Figure 1B**), starch accumulation in leaves is the result of the balance between *de novo* starch synthesis from ADPG entering the chloroplast and breakdown, and the efficiency by which starch breakdown products are recycled back to starch by means of pPGM and AGP. Thus, according to this interpretation of transitory starch biosynthesis, starch is actively synthesized in pPGM and AGP mutants, but its accumulation is prevented due to the blockage of the mechanism of scavenging of glucose units derived from the starch breakdown [15], [34]. The occurrence of starch turnover during illumination is not surprising since pulse-chase and starch-preloading experiments using isolated chloroplasts [35], [36], intact leaves [37], [38], or cultured photosynthetic cells [39] have shown that chloroplasts can synthesize and mobilize starch simultaneously. Furthermore, recent metabolic flux analyses carried out using illuminated Arabidopsis plants cultured in ^13^CO_2_-enriched environment revealed rapid labelling of maltose, the main starch degradation product [40]. Also, leaves of *sex1-1* mutants impaired in β-amylolytic starch breakdown accumulated 3–4 fold more starch than WT leaves when plants were cultured under continuous light conditions [41]. Moreover, simultaneous synthesis and breakdown of glycogen has been shown to widely occur in animals [42]–[44] and in bacteria [45]–[48]. In this respect we must emphasize that many bacterial species co-express glycogen biosynthetic and breakdown genes in a single transcriptional unit, which guarantees simultaneous synthesis and breakdown of glycogen [49]–[53] (for a review see [54]).

The possible occurrence of sources, other than the pPGI-pPGM-AGP pathway, of ADPG linked to starch biosynthesis has been a matter of debate for more than 20 years [3]–[5], [28]–[30], [32], [34], [55]–[60]. In attempting to solve this controversy, we recently carried out HPLC analyses of ADPG content in leaves of the near-starchless *adg1-1* and *aps1* Arabidopsis mutants impaired in AGP [15]. We also measured the ADPG content in the leaves of both wild type (WT) and *aps1* plants ectopically expressing the *Escherichia coli* ADPG hydrolase EcASPP [61] either in the cytosol or the chloroplast [15]. We reasoned that if leaves produce starch from ADPG exclusively synthesized in the plastid, plastidial expression of EcASPP competing with SS for ADPG, but not cytosolic EcASPP expression, should lead to reduction of both starch and ADPG content. Conversely, if ADPG linked to starch biosynthesis occurs both in the plastid and in the cytosol, but mainly accumulates in the cytosol, plants expressing EcASPP in the cytosol should accumulate reduced levels of both ADPG and starch content, whereas plants expressing EcASPP in the plastid should accumulate normal ADPG but reduced starch. We also measured the starch and ADPG contents in leaves of *aps1* mutants expressing in the chloroplast the *E. coli* AGP (GlgC) [15]. We found that *adg1-1* and *aps1* leaves accumulate nearly WT ADPG contents, the estimated values of ca. 0.3–0.4 nmol ADPG/g fresh weight (FW) being comparable to those reported by Szecowka et al. [40], Barratt et al. [58] and Crumpton-Taylor et al. (Table S3 in [62]) for WT leaves using HPLC-MS/MS, and those reported by Chen and Thelen [63] using HPLC. These values, however, were 5–10 fold lower than those reported for WT leaves by Lunn et al. [64], Ragel et al. [65], Martins et al. [66] and Crumpton-Taylor et al. (Table 1 in [62]) using HPLC-MS/MS. We also found that *aps1* leaves expressing GlgC in the plastid accumulate WT levels of both starch and ADPG [15]. As expected, expression of EcASPP in the chloroplast resulted in the reduction of starch content [15]. Noteworthy, this reduction in starch content was not accompanied by a significant reduction in the intracellular levels of ADPG. Moreover, plants expressing EcASPP in the cytosol accumulated reduced levels of both starch and ADPG [15]. The overall data thus provided strong evidence that (a) there occur important source(s) other than AGP, of ADPG linked to starch biosynthesis, (b) AGP is a major determinant of starch accumulation but not of intracellular ADPG content in Arabidopsis, (c) most of ADPG has an extraplastidial localization in WT leaves and (d) cytosolic ADPG is linked to starch biosynthesis. The occurrence of an important pool of cytosolic ADPG is not surprising since leaf cells possess cytosolic ADPG metabolizing enzymes such as ADPG phosphorylase (ADPGP) [67] and glucan synthase [68]. Likewise, low steady state concentration of ADPG in the plastid should be expected since (a) this nucleotide-sugar spontaneously hydrolyzes to AMP and glucose1,2-monophosphate under conditions of alkaline pH and high Mg^2+^ concentration occurring during starch biosynthesis in the illuminated chloroplast [56], [69], and (b) SSs rapidly remove ADPG from the stroma to produce starch.

Our previous HPLC analyses of ADPG content in Arabidopsis leaves have been recently questioned by Stitt and Zeeman [5] and Ragel et al. [65], who used HPLC-MS/MS to measure ADPG content in Arabidopsis leaves. These authors reported that *aps1* leaves and leaves of *pgm* plants impaired in pPGM accumulate far lower levels of ADPG than WT leaves. However, values of ADPG content in *pgm* and *aps1* leaves were not shown [5] or not clearly presented [65]. Subcellular localization and determination of ADPG content is critically important to understand starch metabolism and its regulation and connection with other metabolic pathways. Thus, to further investigate the possible occurrence of important ADPG biosynthetic pathway(s) alternative to pPGI-pPGM-AGP, and to test the validity of our previous HPLC-based results and conclusions on ADPG content and subcellular localization, in this work two independent laboratories have carried out HPLC-MS/MS based analysis of ADPG content in leaves of the near-starchless *pgm, aps1* and double *pgm/aps1* mutants cultured under two different conditions. We also measured the ADPG content in WT and *aps1* leaves ectopically expressing in the plastid two different ADPG cleaving enzymes, and in *aps1* leaves expressing GlgC in the plastid. Furthermore, we measured the ADPG content in *ss3/ss4/aps1* mutants impaired in starch granule initiation and chloroplastic ADPG synthesis. We found that *pgm, aps1* and *pgm/aps1* leaves, and leaves with reduced starch content as a consequence of the ectopic expression of ADPG breakdown enzymes in the plastid accumulate nearly WT ADPG content. Furthermore, we found that *aps1* leaves ectopically expressing GlgC in the plastid accumulate WT starch and ADPG contents. In clear contrast, *ss3/ss4/aps1* leaves accumulated ca. 300 fold-more ADPG than WT leaves. The overall data showed that, in Arabidopsis leaves, (a) there are important ADPG sources other than the pPGI-pPGM-AGP pathway, (b) pPGM and AGP are not major determinants of intracellular ADPG content, and (c) the contribution of the chloroplastic ADPG pool to the total ADPG pool is low.

## Materials and Methods

### Plants, bacterial strains and plant transformation

The work was carried out using WT *Arabidopsis* (ecotype Columbia), the *aps1::T-DNA* mutant (SALK_040155) [13], the double *aps1::T-DNA/pgm::T-DNA* mutant, the double *ss3::T-DNA/ss4::T-DNA* mutant [70], the *pgm::T-DNA* mutant (GABI_094D07), the triple *ss3::T-DNA/ss4::T-DNA/aps1::T-DNA* mutant as well as WT, *aps1* and *ss3/ss4* plants transformed with either *35S-TP-P541-glgC* [15], *35S-TP-P541-EcASPP* [15], [27], *35S-TP-P541-AtADPGP* or *35S-TP-P541-AtADPGP-GFP* (this work, see below). Plants were grown in pots either on soil or solid MS medium at ambient CO_2_ in growth chambers at 20°C under a 16 h light (90 µmol photons sec^−1^ m^−2^)/8 h dark regime.

Triple s*s3/ss4/aps1* and double *aps1/pgm* mutants were obtained by crossing and selecting mutants from segregating the F2 populations by PCR on genomic DNA, using the oligonucleotide primers listed in **Table S1 and Table S2**, respectively. Different *35S-TP-P541-AtADPGP, 35S-TP-P541-EcASPP* and *35S-TP-P541-AtADPGP-GFP* plasmid constructs conferring resistance to either kanamycin or hygromycin were produced as illustrated in **Figure S1**. Constructs conferring resistance to hygromycin were used to transform *ss3/ss4* plants. Plasmid constructs were electroporated and propagated in *E. coli* TOP 10. Transfer of the plasmid construct to *Agrobacterium tumefaciens* EHA105 cells was carried out by electroporation. Transformation of *Arabidopsis* plants were conducted as described by Clough and Bent [71]. Transgenic plants were selected on the adequate (kanamycin- or hygromicin-containing) selection medium.

### Enzyme assays

Leaves of 4-weeks old plants were harvested, freeze-clamped and ground to a fine powder in liquid nitrogen with a pestle and mortar. One g of the frozen powder was resuspended at 4°C in 5 ml of 100 mM HEPES (pH 7.5), 2 mM EDTA and 5 mM dithiothreitol, and desalted by ultrafiltration on Centricon YM-10 (Amicon, Bedford, MA). The proteins retained in the filter then were resuspended in 100 mM HEPES (pH 7.5), 2 mM EDTA and 5 mM dithiothreitol. ADPG hydrolytic activity was assayed using the two-step spectrophotometric determination of G1P described by Rodríguez-López et al. [72]. ADPGP activity was assayed at 37°C in the direction of ADPG breakdown in two steps: (1) ADPGP reaction, and (2) measurement of G1P. In step one, the ADPGP assay mixture contained 50 mM HEPES (pH 7.0), 1 mM ADPG, 2 mM Pi, 1 mM MgCl_2_, 1 mM dithiothreitol and the leaf extract in a total volume of 50 µl. The reaction was initiated by adding the leaf extract to the assay mixture. All assays were run with minus ADPG blanks. After 3 min at 37°C, reactions were stopped by boiling the assay reaction mixture for 2 min. In step two, G1P formed was determined spectrophotometrically in a 300 µl mixture containing 50 mM HEPES (pH 7.0), 1 mM EDTA, 2 mM MgCl_2_, 15 mM KCl, 0.6 mM NAD^+^, 1 unit (U) each of PGM and G6P dehydrogenase from *Leuconostoc mesenteroides,* and 30 µl of the step-one reaction. We define 1 U of enzyme activity as the amount of enzyme that catalyzes the production of 1 µmol of product per min.

### Production of polyclonal antisera against AtADPGP and western blot analyses

A complete AtADPGP encoding cDNA from the Arabidopsis Biological Center at Ohio State University [73] was cloned into the pDEST17 expression vector (Invitrogen) to create pDEST17-AtADPGP (**Figure S2**). BL21 C43 (DE3) cells transformed with pDEST-AtADPGP were grown in 100 ml of liquid LB medium to an absorbance at 600 nm of 0.5 and then 1 mM IPTG was added. After 5 h, cells were centrifuged at 6,000 g for 10 min. The pelleted bacteria were resuspended in 6 ml of His-bind binding buffer (Novagen), sonicated and centrifuged at 10,000 g for 10 min. The supernatant thus obtained was subjected to His-bind chromatography (Novagen). The eluted His-tagged AtADPGP was then rapidly desalted by ultrafiltration on Centricon YM-10 (Amicon, Bedford, MA).

The purified recombinant AtADPGP was electrophoretically separated by 12% SDS-PAGE and stained with Coomassie Blue. A ca. 38 kDa protein band was eluted and utilized to produce polyclonal antisera by immunizing rabbits.

For immunoblot analyses, samples were separated on 10% SDS-PAGE, transferred to nitrocellulose filters, and immunodecorated by using the antisera raised against either AtADPGP or EcASPP [27] as primary antibody, and a goat anti-rabbit IgG alkaline phosphatase conjugate (Sigma) as secondary antibody.

### Assay of ADPG content by HPLC-MS/MS

Fully expanded source leaves of 4-weeks old plants were harvested at the indicated illumination period, freeze-clamped and ground to a fine powder in liquid nitrogen with a pestle and mortar. ADPG was then immediately extracted as described by Lunn *et al.* [64]. Aliquots (50–100 mg FW) of the frozen powdered leaves were transferred to pre-cooled tubes and quenched by adding 250 µL of ice-cold CHCl_3_/CH_3_OH (3∶7, v/v). The frozen mixture was warmed to −20°C with vigorous shaking, and incubated at −20°C for 2 h. ADPG was extracted from the CHCl_3_ phase by adding 200 µL of water and warming to 4°C with repeated shaking. After centrifugation at 420 g for 4 min, the upper, aqueous-CH_3_OH phase was transferred to a new tube, and kept at 4°C. The lower, CHCl_3_ phase was re-extracted with 200 µL of cold water, centrifuged, and the second aqueous-CH_3_OH extract was added to the first. The combined aqueous-CH_3_OH extract was freeze-dried using a lyophilizer and re-dissolved in 250 µL of water. High molecular-mass components were removed from the samples by ultrafiltration on vivaspin 500 centrifugal concentrator (Sartorius) at 2,300 g for 2–3 h, 20°C. Recovery experiments were carried out by the addition of known amounts of ADPG disodium salt (Sigma-Aldrich A0627) standards to the frozen tissue slurry immediately after addition of the cold CHCl_3_/CH_3_OH. As described in the “Results and Discussion” section, we found endogenous levels of ADPG of ca. 0.13±0.03 nmol ADPG/g FW (equivalent to 3.1 pmol per 100 µL of extract) in leaves of plants cultured on soil. We thus added 5, 10 or 20 pmol of authenticated ADPG standard to the 50–100 mg samples of the frozen plant material (containing 6.5–13 pmol of ADPG) before extraction. Recoveries of the added 5, 10, and 20 pmol of ADPG were 94±5.3, 93±4.9 and 89±6.6, respectively, demonstrating that even the smallest amounts of ADPG were quantitatively recovered.

ADPG content in leaves of plants cultured on soil was measured in the Research Support Service at the Public University of Navarra using an Agilent 1100 HPLC fitted with a Xbridge C18 column (100×3.0 mm I.D. particle size 3.5 µm) coupled to a MSD-Trap spectrometer (Agilent). The column was equilibrated with a mixture of 99% solution A (15 mM acetic acid and 10 mM triethylamine, pH 4.95) and 1% solution B (methanol) for 7 min before each sample run. The extracts were eluted with a multi-step gradient as follows: 0–4 min, 99% A; 4–25 min, 99–10% A; 25–30 min, 10–10% A; 30–35 min, 10–95% A. ADPG peak detection in the HPLC elute was made after entering directly into the MSD-Trap, which was operated in a multiple reaction monitoring mode, with an electrospray ionization source in negative ionization mode. For ADPG measurement the parent and product ions selected were 587.8 m/z and 346.1 m/z, respectively, and the fragmentation amplitude was 2.0. ADPG was quantified by comparison of the integrated MSD-Trap signal peak area with a calibration curve obtained using ADPG disodium salt as standard.

ADPG of leaves of plants cultured on solid MS was extracted as described above and measured in Niigata University using liquid chromatography-mass spectrometer consisting of LaChrom Elite-HPLC system with L-2130 pump (Hitachi) and LTQ Orbitrap XL (ThermoFisher Scientific) controlled by Xcalibur 2.0 software as described previously [74]. Reversed-phase ion-pair chromatography separation was carried out on a Hypersil GOLD column (50×2.1 mm, 5 µm particle size, ThermoFisher Scientific). An aliquot of sample (10 µl) was loaded onto the Hypersil GOLD column equilibrated with solvent A at flow rate of 150 µl min^−1^. Solvent A was 97∶3 water:methanol with 10 mM tributylamine and 15 mM acetic acid; solvent B was methanol. The gradient is: 0–2.5 min, 100% A; 2.5–5 min, 100–80% A; 5–7.5 min, 80% A; 7.5–13 min, 80–45% A; 13–15.5 min, 45–5% A; 15.5–18.5 min, 5% A; 18.5–19 min, 5–100% A; 19–25 min, 100% A. Other liquid chromatography parameters are autosampler temperature 4°C, injection volume 10 µl, and column temperature 30°C. The MS data was acquired full scans from 450–1000 m/z at 1 Hz and 30,000 resolution in negative ion mode using only Orbitrap.

### Confocal microscopy

Subcellular localization of AtADPGP-GFP was performed using D-Eclipse C1 confocal microscope (NIKON, Japan) equipped with standard Ar 488 laser excitation, BA515/30 filter for green emission, BA650LP filter for red emission and transmitted light detector for bright field images.

### Starch measurement

Starch was measured by using an amyloglucosydase–based test kit (Boehringer Manheim).

## Results and Discussion

### *pgm* and *aps1* leaves accumulate WT ADPG content

**Figure 2:**
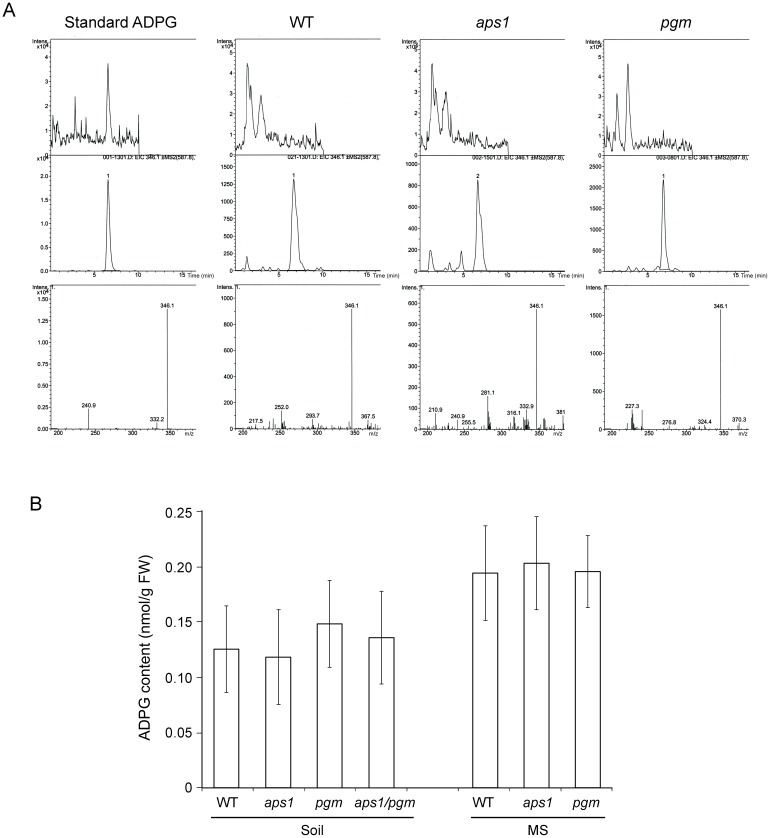
*aps1* and *pgm* leaves accumulate WT ADPG content. (A) HPLC-MS/MS detection of ADPG in WT, *aps1* and *pgm* leaves. Upper panels: Total ion chromatograms (TIC) of extracts from the indicated plants in which the selected fragmentation parent ion was 587.8 m/z. Middle panels: Extracted ion chromatograms (EIC) in which the selected ion for fragmentation of the parent ion was 346.1 m/z. Lower panels: Mass spectra (MS2) obtained from fragmentation of parent ion. ADPG was measured using an Agilent 1100 HPLC fitted with a Xbridge C18 column (100×3.0 mm I.D. particle size 3.5 µm) coupled to a MSD-Trap spectrometer (Agilent) (see Materials and Methods for further details). (B) ADPG content in WT, *aps1, pgm* and *aps1/pgm* leaves. Plants were simultaneously grown either in soil or solid MS. Leaves from 4-weeks old WT, *aps1, pgm* and *aps1/pgm* plants were simultaneously harvested after 10 h of illumination. ADPG was simultaneouly extracted from leaves of WT, *aps1, pgm* and *aps1/pgm* plants and content was simultaneously measured by HPLC-MS/MS as described in Materials and Methods. Note that, consistent with [15], leaves of *aps1, pgm* and *aps1/pgm* plants accumulated WT ADPG content. Values represent the mean ±SD of determinations on three independent samples.

We conducted HPLC-MS/MS analyses of ADPG content in *pgm* and *aps1* mutants cultured on soil and solid MS medium conditions (see Materials and Methods for further details). As shown in **Figure 2**, these analyses revealed that ADPG contents in leaves of WT plants cultured on soil and solid MS medium after 10 h of illumination were 0.13±0.03 and 0.19±0.05 nmol ADPG/g FW, respectively. These values were comparable to those of previous HPLC and HPLC-MS/MS analyses of ADPG content [15], [31], [58], [63]. Most importantly, these analyses also revealed that, irrespective of the culture conditions, ADPG contents in *pgm* and *aps1* leaves were comparable to those of WT leaves (**Figure 2B**). Moreover, leaves of WT, *pgm* and *aps1* cultured on soil accumulated ca. 0.015 nmol ADPG/g FW in the end of the dark period (not shown).

Leaves impaired in pPGM and AGP accumulate 0.5%–3% of the WT starch [4], [14], [15], [17], [75]. The occurrence of low starch content in *pgm* leaves has been ascribed to marginally low import to the chloroplast of cytosolic G1P and subsequent AGP-mediated conversion into ADPG [8], [57], [65], [75], whereas the occurrence of reduced starch content in *aps1* leaves has been ascribed to residual AGP activity from the large subunits [5], [65]. The latter explanation is highly unlikely, since the AGP large subunits are unstable in the absence of APS1 in Arabidopsis [14], [15]. Therefore, *aps1* null mutants lack not only the small APS1 AGP subunits, but also the large AGP subunits [14], [15], which results in total lack of AGP activity [13], [15].

We reasoned that if the above interpretations for the occurrence of reduced starch content in *aps1* and *pgm* leaves were correct, and if the pPGI-pPGM-AGP pathway is the sole source of ADPG in leaves, leaves of double *aps1/pgm* mutants should not accumulate any starch and ADPG at all. To test this hypothesis we crossed *aps1* and *pgm* mutants as indicated in Materials and Methods. The resulting *aps1/pgm* mutants were cultured on soil and the leaf ADPG and starch contents were measured. As shown in **Figure 2B**, this analyses revealed that, similar to *pgm* and *aps1* leaves, *aps1/pgm* leaves accumulated nearly WT ADPG content (**Figure 2B**). Furthermore, leaves of the double *aps1/pgm* mutant accumulated ca. 1.5% of the WT starch content.

The overall data (a) showed that pPGM and AGP are not major determinants of intracellular ADPG content, and (b) were consistent with the occurrence in Arabidopsis leaves of important ADPG source(s) other than the pPGI-pPGM-AGP pathway.

### The contribution of the chloroplastic ADPG pool to the total ADPG pool is low in WT and *aps1* leaves

**Figure 3:**
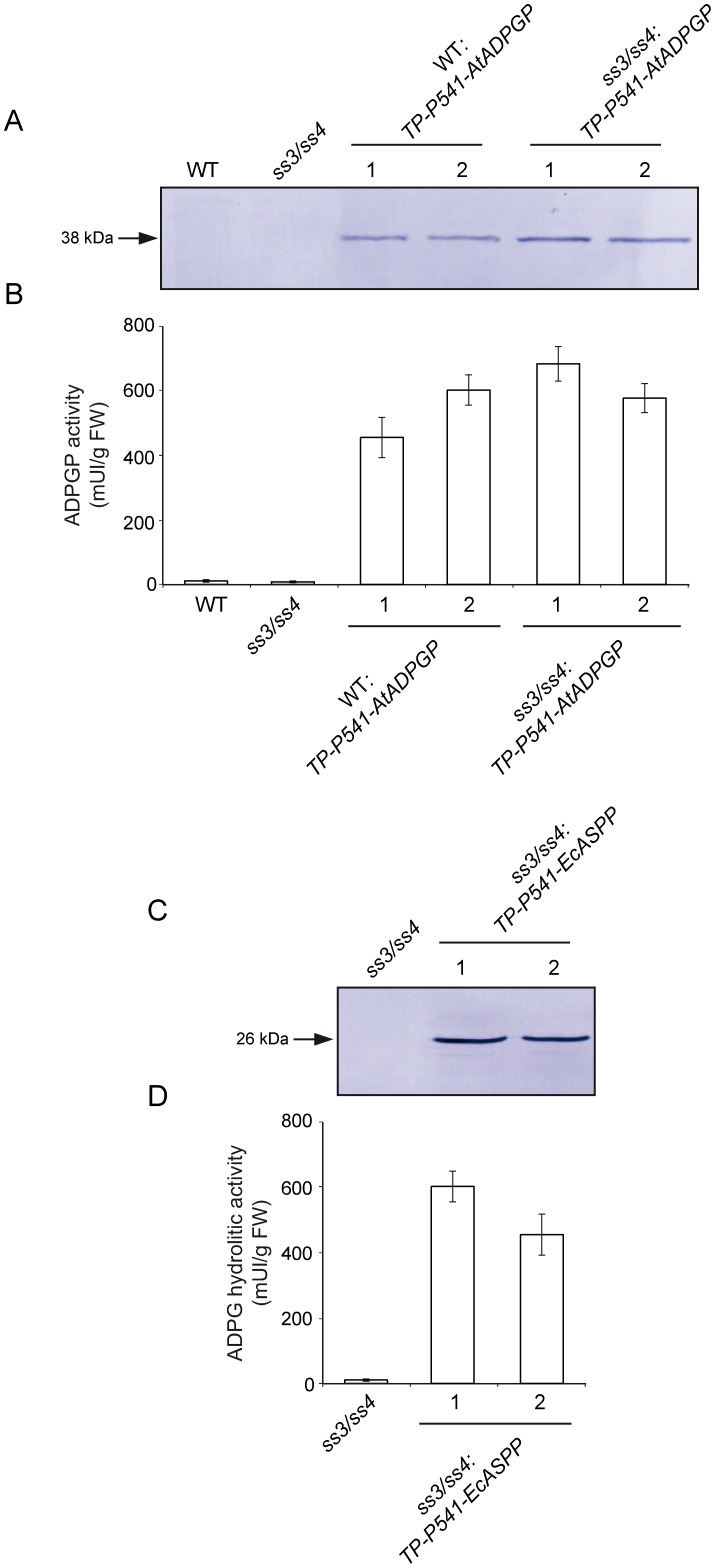
Production of WT and *ss3/ss4* plants expressing AtADPGP or EcASPP in the plastid. (A) Western blot of AtADPGP in leaves of WT and *ss3/ss4* plants, and leaves of two independent lines each of *TP-P541-AtADPGP* expressing WT plants and *TP-P541-AtADPGP* expressing *ss3/ss4* plants. (B) ADPGP activity in leaves of WT and *ss3/ss4* plants, and leaves of *TP-P541-AtADPGP* expressing WT plants and *TP-P541-AtADPGP* expressing *ss3/ss4* plants. (C) Western blot of EcASPP in *ss3/ss4* leaves, and leaves of two independent lines of *TP-P541-EcASPP* expressing *ss3/ss4* plants. (D) ASPP activity in *ss3/ss4* leaves and leaves of two independent *TP-P541-EcASPP* expressing *ss3/ss4* plants. In “A” and “C”, the gels were loaded with 20 µg per lane of protein and AtADPGP and EcASPP were immunodecorated by using antisera specifically raised against AtADPGP and EcASPP.

Our previous HPLC based ADPG content measurement analyses revealed that *aps1* leaves expressing in the plastid either GlgC (*TP-P541-glgC* expressing *aps1* plants) or EcASPP (*TP-P541-EcASPP* expressing *aps1* plants) accumulate WT ADPG content [15]. *TP-P541-glgC* expressing *aps1* leaves accumulated WT starch content, whereas *TP-P541-EcASPP* expressing *aps1* leaves accumulated ca. 50% less starch than *aps1* leaves [15]. The overall data thus provided evidence that (a) AGP is not a major determinant of intracellular ADPG levels, and (b) the contribution of plastidic ADPG to the total ADPG pool is very low in *Arabidopsis* leaves [15]. To further test the validity of these conclusions, we measured by HPLC-MS/MS the ADPG content in leaves of two independent *TP-P541-glgC* expressing *aps1* lines and two independent *TP-P541-EcASPP* expressing *aps1* and WT plants. We must emphasize that our previous studies showed that leaves of *TP-P541-EcASPP* expressing plants accumulate as much as ca. 50% of the starch accumulated by the leaves of the parental plants [15], [27]. This moderate reduction in the starch content exerted by the ectopic expression of EcASPP in the chloroplast may be ascribed to the relatively low affinity of EcASPP for ADPG (K_m_ being 160 µM [61]) combined with very low concentration of ADPG in the chloroplast. Therefore, to further reduce the plastidic ADPG pool we also produced WT plants expressing *Arabidopsis thaliana* ADPG phosphorylase (AtADPGP) in the chloroplast (*TP-P541-AtADPGP* expressing WT plants) (**Figure 3A,B**). ADPGP (E.C. 2.7.7.36) is a widely distributed cytosolic enzyme exhibiting high affinity for ADPG (K_m_ for ADPG being 7 µM [67]) that catalyzes the phosphorolytic breakdown of ADPG into ADP and G1P [67], [76], [77]. Fluorescence distribution pattern in TP-P541-AtADPGP-GFP expressing cells confirmed the exclusive plastidial localization of AtADPGP in *TP-P541-AtADPGP* expressing cells (**Figure S3**).

**Figure 4:**
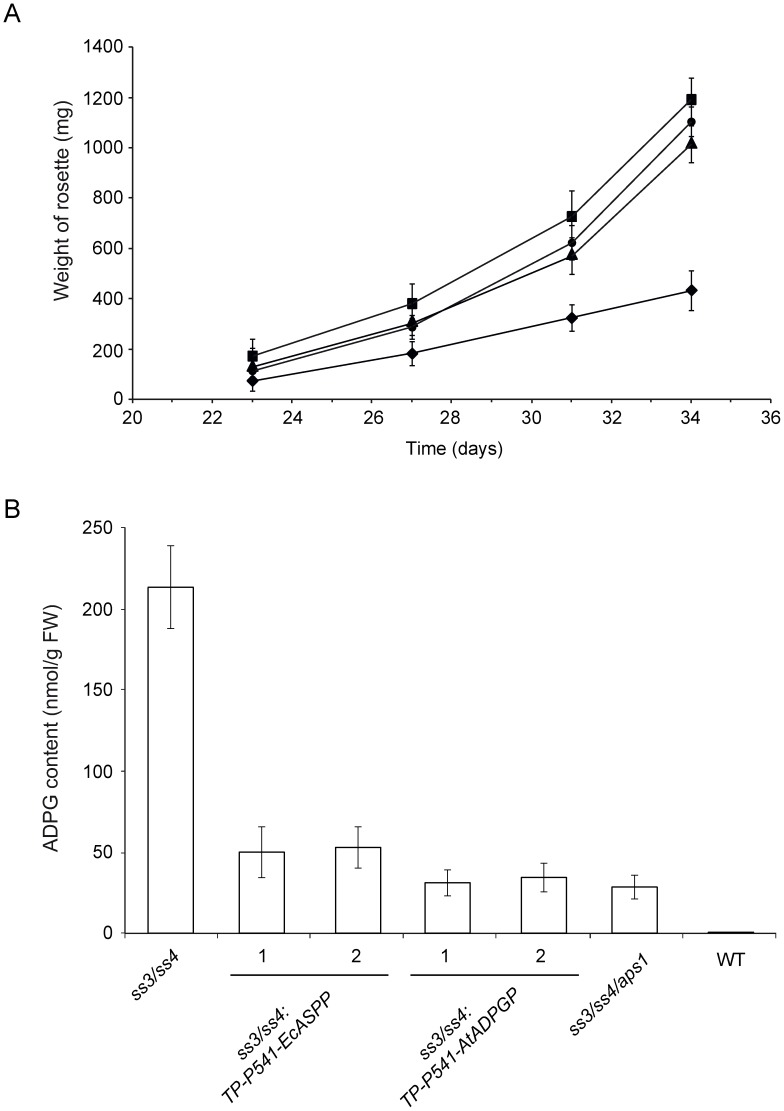
Ectopic expression of EcASPP and AtADPGP in the plastid restores the WT growth and partially reverts the ADPG excess phenotype of *ss3/ss4* plants. (A) Time-course of fresh weight of rosettes of WT (▪) and *ss3/ss4* (⧫) plants, and rosettes of one representative line each of *TP-P541-AtADPGP* expressing *ss3/ss4* plants and *TP-P541-EcASPP* expressing *ss3/ss4* plants (• and ▴, respectively). Plants were grown under long-day conditions (16 h light/8 h dark, 20°C) and at an irradiance of 90 µmol photons sec^−1^ m^−2^. Values represent the mean ±SD of determinations on five independent plants. (B) ADPG content in WT, *aps1* and *ss3/ss4* leaves, and leaves of plants of two independent *TP-P541-EcASPP-* and *TP-P541-AtADPGP-* expressing *ss3/ss4* lines. Leaves were harvested after 10 h of illumination. Values represent the mean ±SD of determinations on three independent samples.

To test whether EcASPP and AtADPGP are active in the chloroplast, we also produced *ss3/ss4* plants ectopically expressing in the plastid either EcASPP or AtADPGP (**Figure 3A–D**). *ss3/ss4* leaves display a high ADPG content phenotype as a consequence of impairments in starch granule initiation and synthesis [65], a phenotype that can be partially reverted by the introduction of the *aps1* mutation [65]. Furthermore, *ss3/ss4* plants display a severe dwarf phenotype due to accumulation of high ADPG content in the chloroplast [65]. We reasoned that if EcASPP and AtADPGP are active in the chloroplast *TP-P541-EcASPP* and *TP-P541-AtADPGP* expressing *ss3/ss4* plants should display a WT growth phenotype and their leaves should accumulate less ADPG than *ss3/ss4* leaves. Consistent with this presumption *TP-P541-EcASPP* and *TP-P541-AtADPGP* expressing *ss3/ss4* plants exhibited a nearly WT growth phenotype (**Figure 4A**). Moreover, the ectopic expression in the plastid of EcASPP and AtADPGP resulted in a 5-fold decrease of ADPG content in *ss3/ss4* leaves (**Figure 4B**). Leaves of *TP-P541-EcASPP* and *TP-P541-AtADPGP* expressing *ss3/ss4* plants still exhibited ca. 300-400 fold more ADPG than WT leaves. This high ADPG content was comparable to that of *ss3/ss4/aps1* leaves impaired in both starch granule initiation and chloroplastic ADPG synthesis (**Figure 4B**). The overall data thus showed that (a) both EcASPP and AtADPGP are active in the chloroplast, and (b) plastidic expression of EcASPP and AtADPGP can be utilized as a trait to reduce the plastidic ADPG pool.

**Figure 5:**
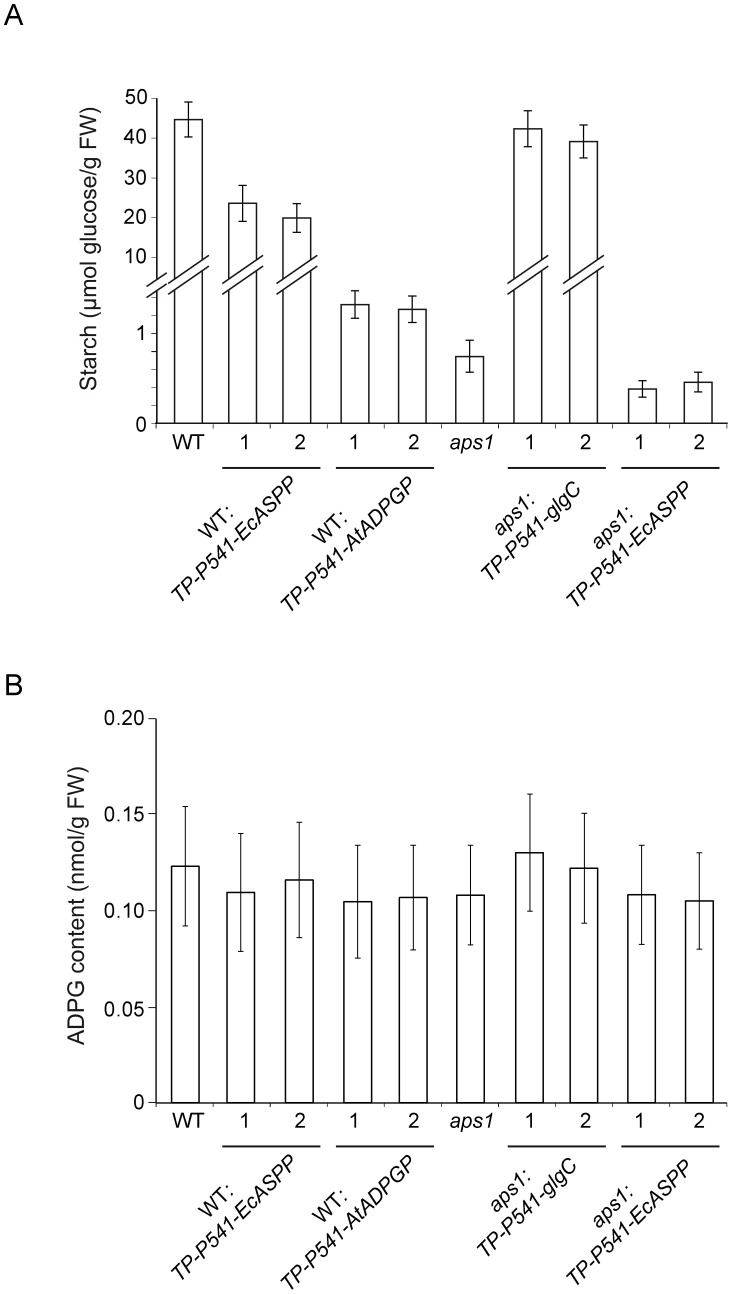
The contribution of the chloroplastic ADPG pool to the total ADPG pool is low in WT and *aps1* leaves. (A) Starch and (B) ADPG contents in leaves of WT and *aps1* plants, and leaves of two independent lines each of *TP-P541-AtADPGP* expressing WT plants, *TP-P541-EcASPP* expressing WT plants, *TP-P541-glgC* expressing *aps1* plants, and *TP-P541-EcASPP* expressing *aps1* plants. Plants were simultaneously grown and leavesf from 4-weeks old plants were simultaneously harvested after 10 h of illumination. ADPG was simultaneouly extracted and measured by HPLC-MS/MS as described in Materials and Methods. Note that, consistent with [15], leaves of *TP-P541-EcASPP* expressing WT plants, *TP-P541-glgC* expressing *aps1* plants, and *TP-P541-EcASPP* expressing *aps1* plants accumulated WT ADPG content. Values represent the mean ±SD of determinations on three independent samples.Values represent the mean ±SD of determinations on three independent samples.

As shown in **Figure 5A**, starch contents in leaves of plants from two independent *TP-P541-AtADPGP* and *TP-P541-EcASPP* expressing WT lines were ca. 3% and 50% of that of WT leaves, respectively, which further confirms that active ADPGP and EcASPP have access to the plastidic pool of ADPG. The fact that the expression of AtADPGP (whose K_m_ for ADPG is 7 µM) in the chloroplast resulted in a strong (ca. 97%) reduction of the starch content, whereas expression of EcASPP (whose K_m_ for ADPG is 160 µM) in the chloroplast resulted in a moderate (ca. 50%) reduction of starch content [15], [27] (**Figure 5A**) points to the occurrence of very low concentration of ADPG in the chloroplast. In line with this presumption, despite the considerable reduction of starch content exerted by the ectopic expression of EcASPP and AtADPGP in the chloroplast, *TP-P541-AtADPGP* and *TP-P541-EcASPP* expressing WT leaves accumulated nearly WT ADPG content when plants were cultured on soil and MS medium (**Figure 5B** **, Figure S4**).

*aps1* leaves accumulate 1–2% of the WT starch content, a phenotype that is reverted to WT by the ectopic expression of GlgC in the plastid [15] (**Figure 5A**). Furthermore, *TP-P541-EcASPP* expressing *aps1* leaves accumulate 40–50% of the starch accumulated by *aps1* leaves [15] (**Figure 5A**). Despite the considerable enhancement and reduction of starch content exerted by the ectopic expression of GlgC and EcASPP in the chloroplast of *asp1* leaves, respectively, leaves of plants from two independent *TP-P541-glgC* expressing *aps1* lines, and leaves of plants from two independent *TP-P541-EcASPP* expressing *aps1* lines accumulated nearly WT ADPG content in two different culture conditions (**Figure 5B** **, Figure S4**).

The overall results thus provided strong evidence that (a) most of ADPG accumulates outside the chloroplast, and (b) the contribution of the chloroplastic ADPG pool to the total ADPG pool is low in WT and *aps1* leaves.

### Additional concluding remarks

HPLC-MS/MS studies carried out in this work by two independent laboratories showed that *aps1, pgm* and *aps1/pgm* leaves accumulate WT ADPG content (**Figure 2**), which provides strong evidence that leaves possess important ADPG sources other than the pPGI-pPGM-AGP pathway. As expected, leaves of plants ectopically expressing in the chloroplast either ADPG synthesis or breakdown enzymes accumulated higher and lower starch levels, respectively, than their parental lines (**Figure 5A**). However, these changes in starch content were not accompanied by concomitant changes in ADPG content (**Figure 5B**). This and the fact that the expression of ADPG cleaving enzymes in the cytosol results in reducing levels of both ADPG and starch [15], [27] strongly indicate that (i) most of ADPG accumulates outside the chloroplast, and (ii) a sizable pool of ADPG occurring in the cytosol is linked to starch biosynthesis. That *ss3/ss4/aps1* leaves impaired in both starch granule initiation and AGP-mediated chloroplastic ADPG synthesis accumulate ca. 300-fold more ADPG than WT leaves (**Figure 4**) further supports the idea that ADPG can be synthesized outside the chloroplast.

Using HPLC-MS/MS we have recently shown that leaves of *pgi1* mutants impaired in pPGI accumulate WT ADPG content [78], which further reinforces the idea that leaves possess important ADPG sources other than the pPGI-pPGM-AGP pathway. These studies also showed that the low starch content phenotype of *pgi1* mutants is largely the consequence of combined factors including reduction of photosynthetic activity, rather than the lack of pPGI-mediated flow between the Calvin-Benson cycle and the pPGM-AGP starch biosynthetic pathway [78]. Moreover, our studies showed that *pgi1* leaves of plants exposed for few hours to the action of microbial volatiles can accumulate up to 15-fold more starch than WT leaves [79]. This, and the facts that (a) chloroplasts can incorporate extraplastidial ADPG by means of a yet to be identified transporter and convert it into starch [33], (b) a sizable pool of ADPG accumulates outside the chloroplast [15], [27], [34] (this work), (c) cytosolic enzymes such as SuSy can produce ADPG [30], [69], [80]–[84], and (d) SuSy expresses in the mesophyll cells [85], [86] prompted us to propose the model of transitory starch metabolism similar to that illustrated in **Figure 1B**.

The suggested starch biosynthetic model illustrated in **Figure 1B** involves simultaneous synthesis and breakdown of starch, and the pPGM and AGP-mediated scavenging of the starch breakdown products, thus making up a starch futile cycle. In this respect we must emphasize that many phylogenetically distant bacteria arrange all glycogen synthetic and breakdown genes in a single transcriptional unit, which guarantees simultaneous expression of glycogen synthesis and breakdown enzymes, and scavenging of glycogen breakdown products [49]–[53]. The resulting glycogen futile cycling would entail advantages such as dissipation of excess energy, sensitive regulation and rapid channeling of metabolic intermediates toward various metabolic pathways in response to biochemical needs [47], [48], [87], [88]. Also, many phylogenetically distant bacteria possess various important sources, other than AGP, of ADPG linked to glycogen biosynthesis [89]–[93]. Since glycogen may play relevant roles in the survival of bacteria to sporadic periods of famine, and because the metabolism of this polyglucan is highly interconnected with multiple and important cellular processes [94], [95], it is conceivable that both glycogen futile cycling and redundancy of ADPG sources were selected during bacterial evolution to guarantee the production of glycogen and its connection with other metabolic processes in response to physiological needs imposed by the environment and lifestyle [54]. Like in bacteria, starch is believed to act as a major integrator of the plant metabolic status that accumulates to cope with temporary starvation imposed by the environment [96]. Starch futile cycling may thus entail advantages such as rapid metabolic channeling toward various pathways (such as biosynthesis of fatty acids, OPPP, sulfolipid) [97], [98] in response to physiological and biochemical needs. It is thus conceivable that, similar to bacteria, both redundancy of ADPG sources and starch futile cycling have been selected during plant evolution to warrant starch production and rapid connection of starch metabolism with other metabolic pathways.

## Supporting Information

Figure S1 **Stages to produce the** ***35S-TP-P541-AtADPGP, 35S-TP-P541-Ec-ASPP*** **and** ***35S-TP-P541-AtADPGP-GFP*** **plasmid constructs used to transform** ***Arabidopsis*** **plants.** For AtADPGP constructs, a complete *AtADPGP* cDNA was obtained from the ABRC cDNA collection (C105280). *TP-P541-AtADPGP* were generated by cloning *AtADPGP* in the plasmid pSK TP-P541 which was used as template to generate *35S-TP-P541-AtADPGP* and *35S-TP-P541-AtADPGP* plasmid constructs using the forward 5′-GGGGACAAGTTTGTACAAAAAAGCAGGCTTAATGACGTCACCGAGCCAT-3′ and the reverse 5′- GGGGACCACTTTGTACAAGAAAGCTGGGTATCAAGT-AAGGCTAACTTCCCGC-3′ primers and the Gateway technology (Invitrogen, http://www.invitrogen.com). To produce the *35S-TP-P541-AtADPGP-GFP* plasmid construct the reverse primer 5′-GGGGACCACTTTGTACAAGAAAGCTGGGTAAG-TAAGGCTAACTTCCCGCATAAC-3′ was used to remove the stop codon from *AtADPGP*. DNA sequences of all constructs were confirmed by sequencing.
(PDF)

Figure S2 **Stages to produce the pDEST17-AtADPGP plasmid construct used to transform** ***E. coli*** **cells.**
(PDF)

Figure S3 **Plastidial localization of AtADPGP-GFP in leaves of** ***TP-P541-AtADPGP-GFP*** **expressing WT plants**. The upper panels show that AtADPGP-GFP fluorescence has a plastidial localization in leaf epidermal and mesophyll cells (bar 10 µm). In the middle panel note that AtADPGP-GFP was present among the grana in the central part of the chloroplast, as well as in the grana-free peripheral part of the chloroplast of mesophyll cells (bar 10 µm). The lower panels show a detailed view of plastid stromules in leaf epidermal cells (bar 2 µm). Note that GFP fluorescence labelled long stroma-filled tubular extensions corresponding to plastid stromules.
(EPS)

Figure S4 **ADPG content in leaves of WT plants, and leaves of** ***TP-P541-AtADPGP*** **expressing WT plants (line #1),** ***TP-P541-EcASPP*** **expressing WT plants (line #1), and** ***TP-P541-EcASPP*** **expressing** ***aps1*** **plants (line #1) cultured on solid MS medium**. Leaves were harvested after 10 h of illumination. Values represent the mean ±SD of determinations on three independent samples.
(EPS)

Table S1 **Primers used for the identification of the triple** ***ss3/ss4/aps1*** **mutant plants.**
(DOC)

Table S2 **Primers used for the identification of the double** ***aps1/pgm*** **mutant plants.**
(DOC)
