# Correction: HPLC-MS/MS Analyses Show That the Near-Starchless *aps1* and *pgm* Leaves Accumulate Wild Type Levels of ADPglucose: Further Evidence for the Occurrence of Important ADPglucose Biosynthetic Pathway(s) Alternative to the pPGI-pPGM-AGP Pathway

**DOI:** 10.1371/journal.pone.0121181

**Published:** 2015-03-24

**Authors:** The PLOS ONE Staff

Reference 78 and 79 are incorrectly switched. The information in reference 78 should be in reference 79 and the information in reference 79 should be in reference 78. The correct reference 78 is: Bahaji A, Sánchez-López AM, Muñoz FJ, Baroja-Fernández E, Li J, et al. (2014) Reevaluating the involvement of plastidic phosphoglucoseisomerase in starch biosynthesis in mesophyll cells. XII Plant Molecular Biology Meeting, Cartagena, Spain. The correct reference 79 is: Bahaji A, Sánchez-López AM, Li J, Baroja-Fernández E, Muñoz FJ, et al. (2013) The Calvin-Benson cycle is not directly linked to transitory starch biosynthesis by means of phosphoglucose isomerase in plants exposed to microbial volatiles. XIII Spain-Portugal Congress on Plant Physiology (24–27 July 2013, Lisbon, Portugal).

There is an error in the legend for Fig. 4. Please see the complete, corrected Fig. 4 here.

**Fig 4:**
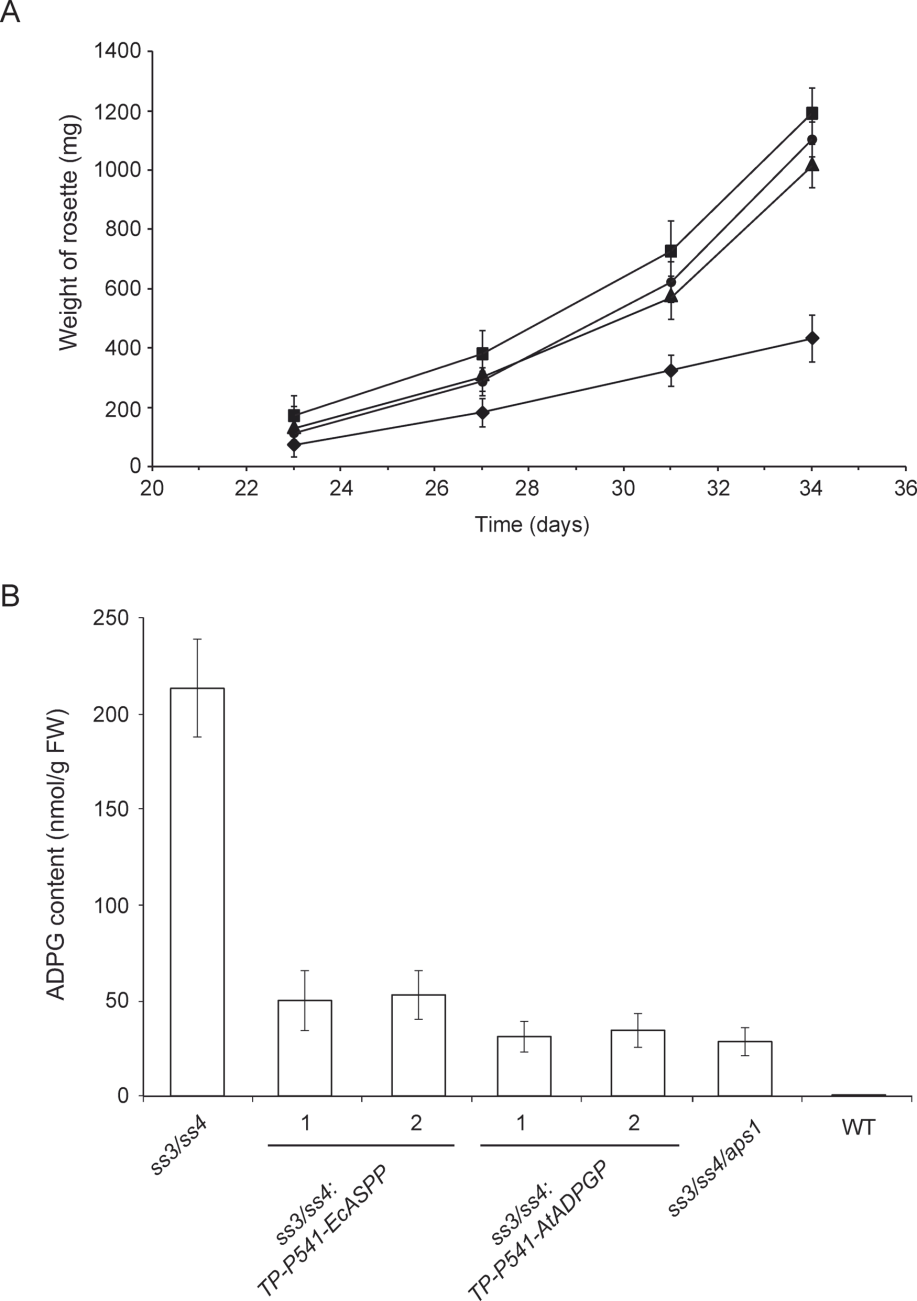
Ectopic expression of EcASPP and AtADPGP in the plastid restores the WT growth and partially reverts the ADPG excess phenotype of *ss3/ss4* plants. (A) Time-course of fresh weight of rosettes of WT (■) and *ss3/ss4* (◆) plants, and rosettes of one representative line each of *TP-P541-AtADPGP* expressing *ss3/ss4* plants and *TP-P541-EcASPP* expressing *ss3/ss4* plants (● and ▲, respectively). Plants were grown under long-day conditions (16 h light/8 h dark, 20°C) and at an irradiance of 90 μmol photons sec^−1^ m^−2^. Values represent the mean ± SD of determinations on five independent plants. (B) ADPG content in WT, ss3/ss4/aps1 and ss3/ss4 leaves, and leaves of plants of two independent *TP-P541-EcASPP-* and *TP-P541-AtADPGP-* expressing *ss3/ss4* lines. Leaves were harvested after 10 h of illumination. Values represent the mean ± SD of determinations on three independent samples.
