# Supplementary figures and images for: HPLC-MS/MS Analyses Show That the Near-Starchless aps1 and pgm Leaves Accumulate Wild Type Levels of ADPglucose: Further Evidence for the Occurrence of Important ADPglucose Biosynthetic Pathway(s) Alternative to the pPGI-pPGM-AGP Pathway
Source: PLoS One. 2014 Aug 18;9(8):e104997. doi: 10.1371/journal.pone.0104997 (PMC4136846; doi:10.1371/journal.pone.0104997)

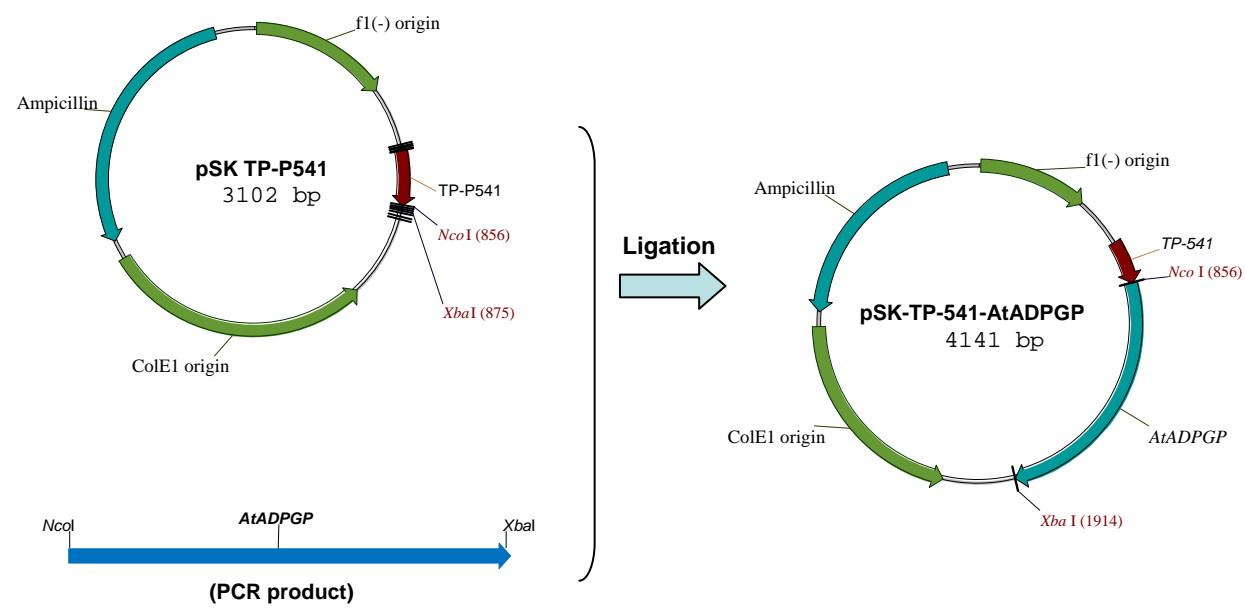

Figure S1

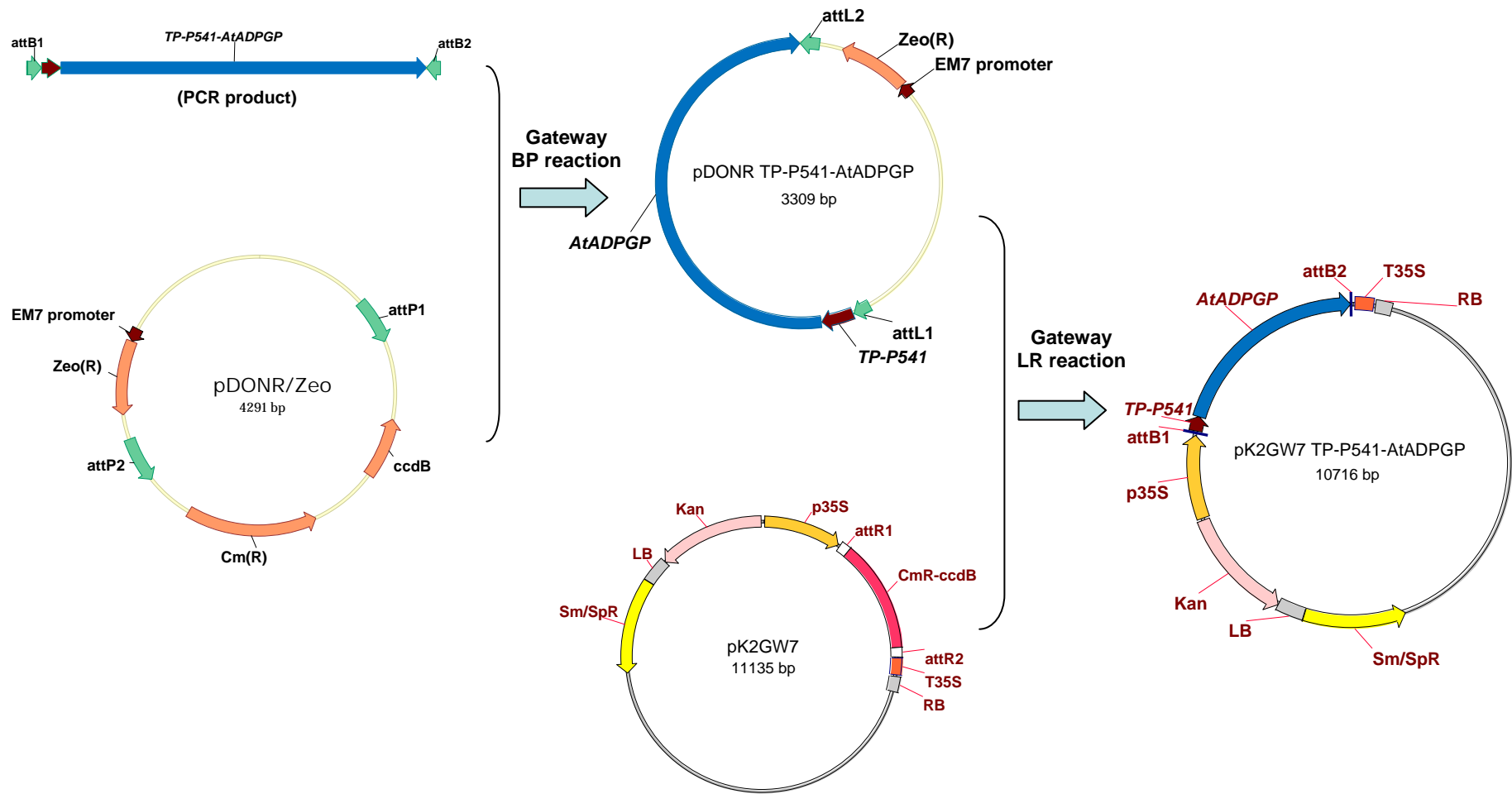

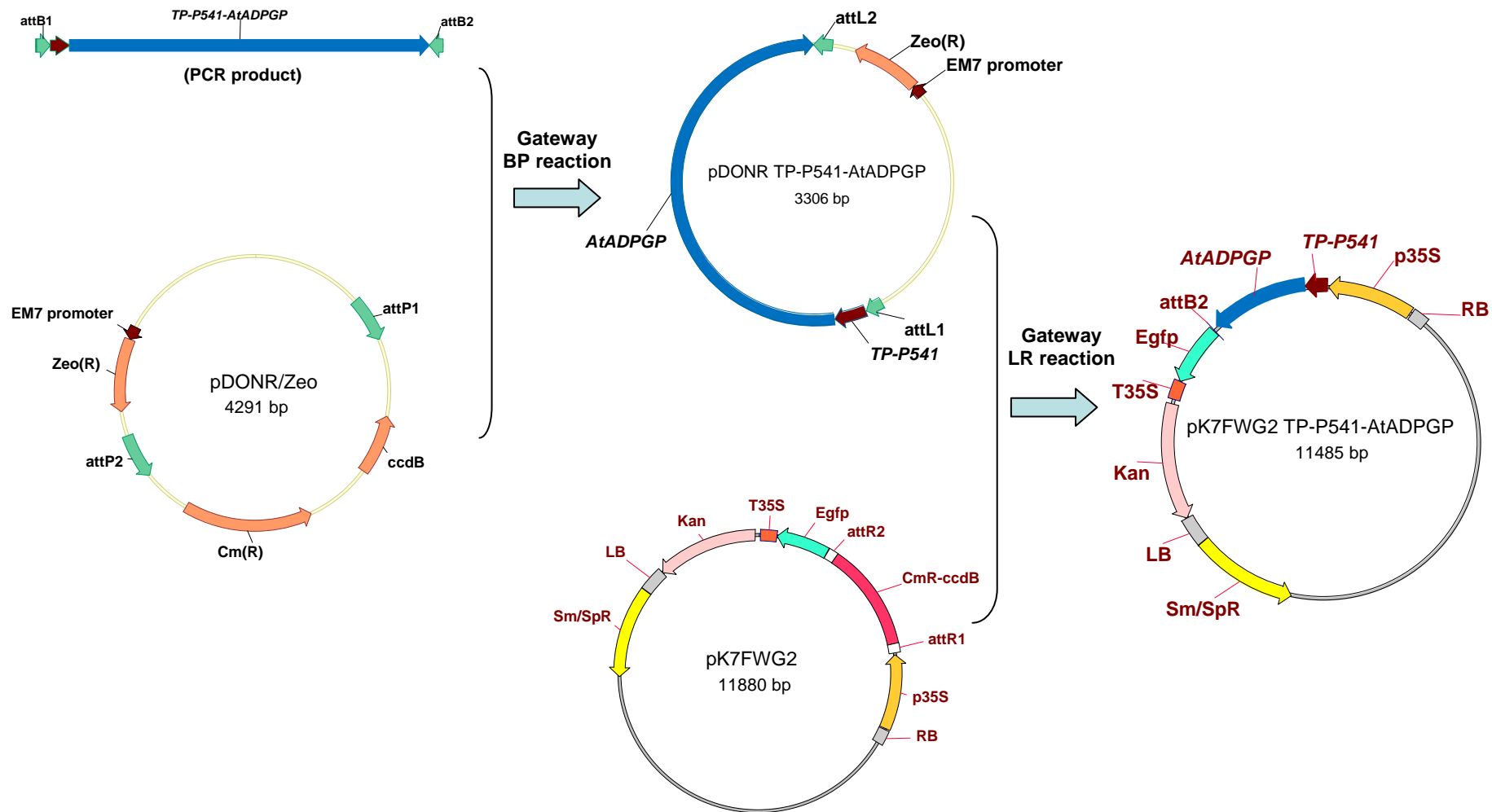

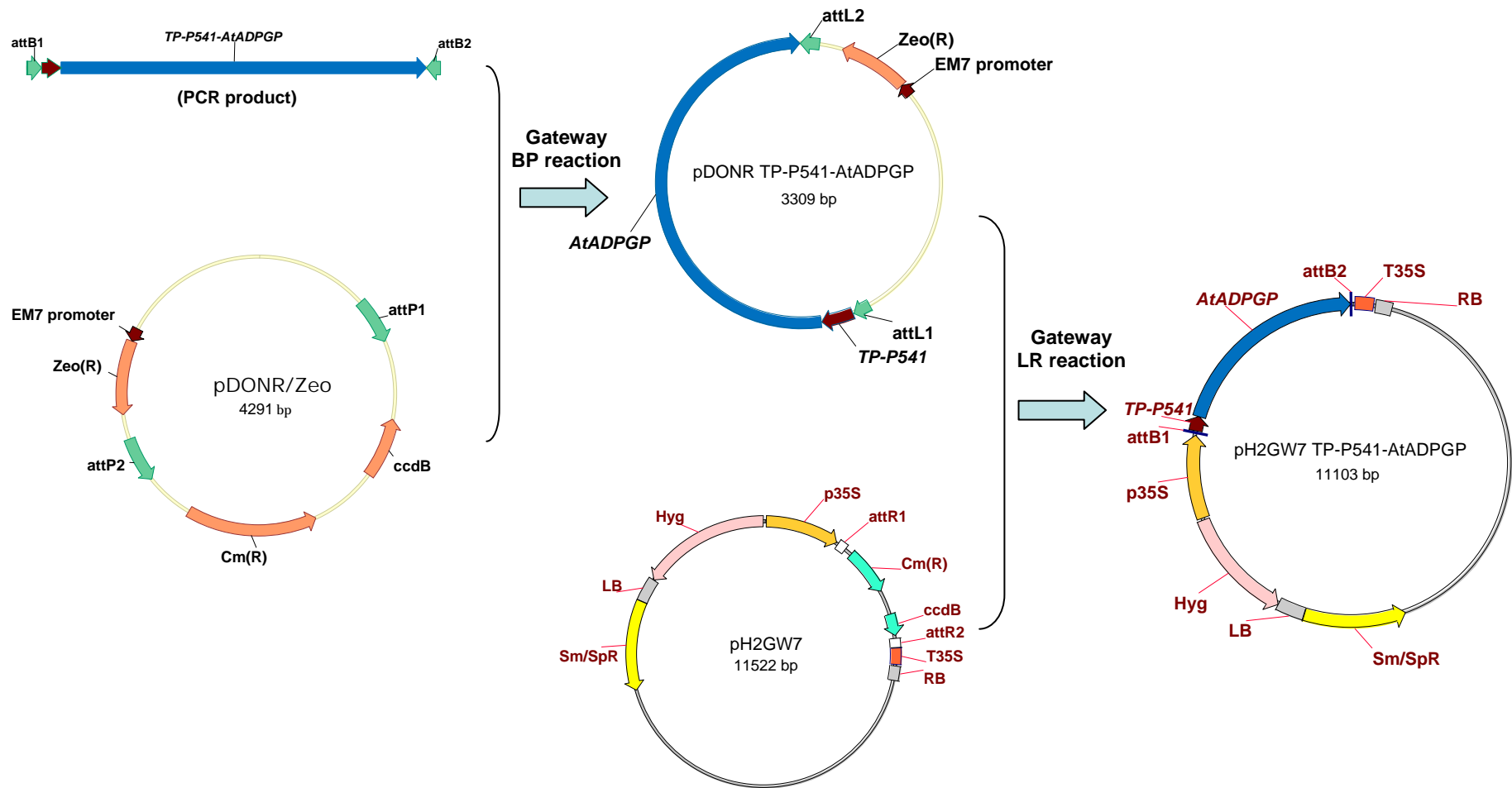

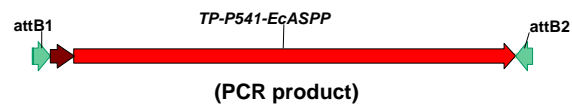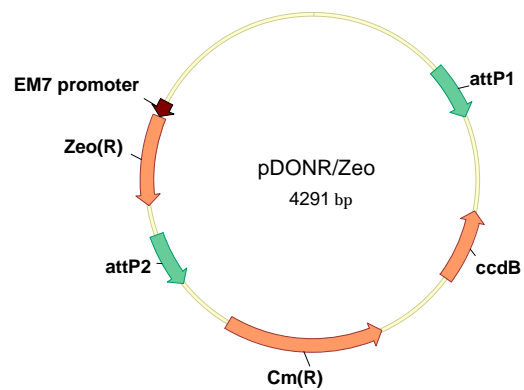

Gateway  
BP reaction

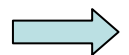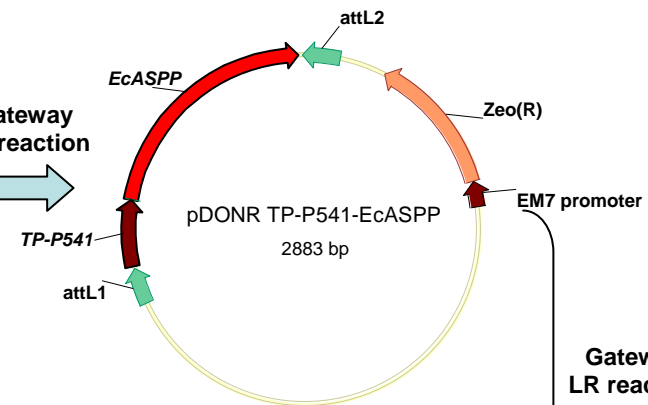

Gateway  
LR reaction

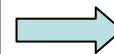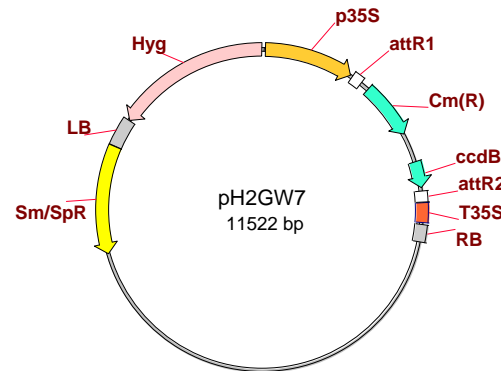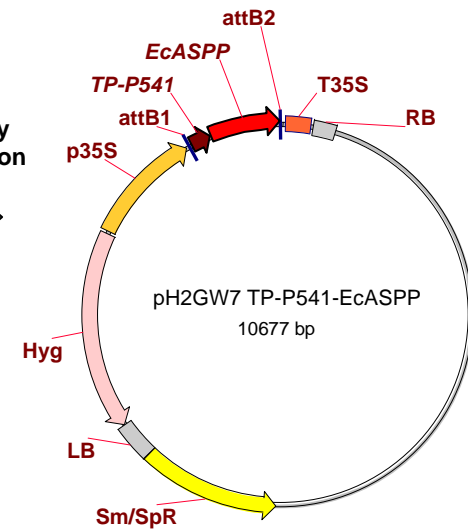

Supplement: Figure S1 — Stages to produce the 35S-TP-P541-AtADPGP, 35S-TP-P541-Ec-ASPP and 35S-TP-P541-AtADPGP-GFP plasmid constructs used to transform Arabidopsis plants. For AtADPGP constructs, a complete AtADPGP cDNA was obtained from the ABRC cDNA collection (C105280). TP-P541-AtADPGP were generated by cloning AtADPGP in the plasmid pSK TP-P541 which was used as template to generate 35S-TP-P541-AtADPGP and 35S-TP-P541-AtADPGP plasmid constructs using the forward 5′-GGGGACAAGTTTGTACAAAAAAGCAGGCTTAATGACGTCACCGAGCCAT-3′ and the reverse 5′- GGGGACCACTTTGTACAAGAAAGCTGGGTATCAAGT-AAGGCTAACTTCCCGC-3′ primers and the Gateway technology (Invitrogen, http://www.invitrogen.com). To produce the 35S-TP-P541-AtADPGP-GFP plasmid construct the reverse primer 5′-GGGGACCACTTTGTACAAGAAAGCTGGGTAAG-TAAGGCTAACTTCCCGCATAAC-3′ was used to remove the stop codon from AtADPGP. DNA sequences of all constructs were confirmed by sequencing. (PDF) [file pone.0104997.s001.pdf]

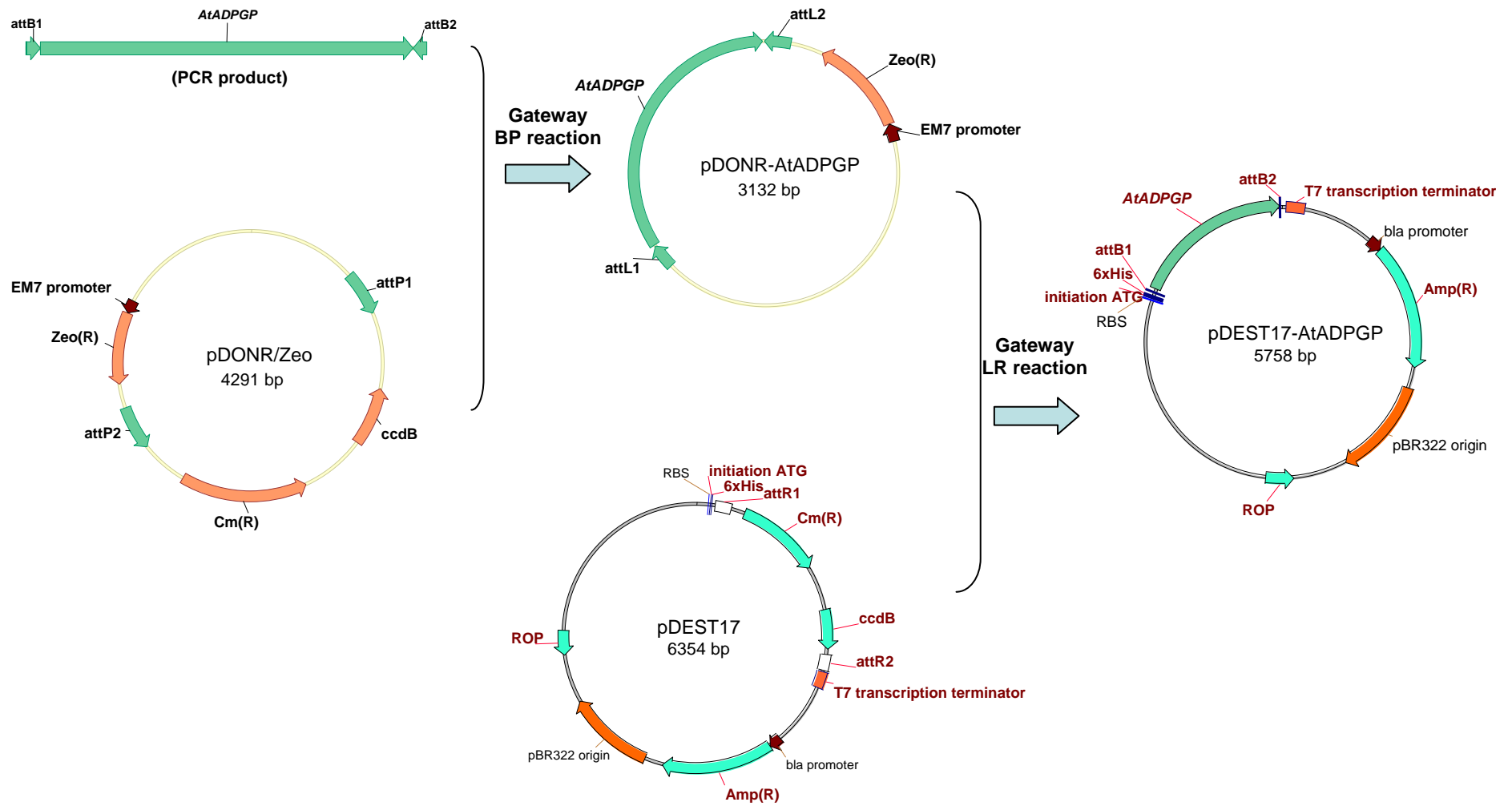

Figure S2

Supplement: Figure S2 — Stages to produce the pDEST17-AtADPGP plasmid construct used to transform E. coli cells. (PDF) [file pone.0104997.s002.pdf]
