# Supplementary material for: HPLC-MS/MS Analyses Show That the Near-Starchless aps1 and pgm Leaves Accumulate Wild Type Levels of ADPglucose: Further Evidence for the Occurrence of Important ADPglucose Biosynthetic Pathway(s) Alternative to the pPGI-pPGM-AGP Pathway
Source: PLoS One. 2014 Aug 18;9(8):e104997. doi: 10.1371/journal.pone.0104997 (PMC4136846; doi:10.1371/journal.pone.0104997)
Supplement: Table S1 — Primers used for the identification of the triple ss3/ss4/aps1 mutant plants. (DOC) [file pone.0104997.s005.doc]

**Table S1:** Primers used for the identification of the triple *ss3/ss4/aps1* mutant plants.

| *APS1* | APS1-LP | 5'-GGTGGTCTTGTCTAGAGTGCAC-3' |
| --- | --- | --- |
|  | APS1-RP | 5'-ACACACAGCCGCGTTATTTACCACCG-3' |
|  | LBb1 T-DNA | 5'-GCGTGGACCGCTTGCTGCAACT-3' |
| *SS3* | SS3-LP | 5´-AGGCTTGGACTAAAGAGTGCC-3' |
|  | SS3-RP | 5´-GCAGAATCCATAGACCTATCACG-3' |
|  | LBb1 T-DNA | 5´-GCGTGGACCGCTTGCTGCAACT-3´ |
| *SS4* | SS4-LP | 5´-GAGCAAGATGATTTCAGACGC-3´ |
|  | SS4-RP | 5´-CAAAGCTCAAAACCACGAAAC-3´ |
|  | GABI T-DNA | 5´-CCCATTTGGACGTGAATGTAGACAC-3´ |
