# Supplementary material for: HPLC-MS/MS Analyses Show That the Near-Starchless aps1 and pgm Leaves Accumulate Wild Type Levels of ADPglucose: Further Evidence for the Occurrence of Important ADPglucose Biosynthetic Pathway(s) Alternative to the pPGI-pPGM-AGP Pathway
Source: PLoS One. 2014 Aug 18;9(8):e104997. doi: 10.1371/journal.pone.0104997 (PMC4136846; doi:10.1371/journal.pone.0104997)
Supplement: Table S2 — Primers used for the identification of the double aps1/pgm mutant plants. (DOC) [file pone.0104997.s006.doc]

**Table S2:** Primers used for the identification of the double *aps1/pgm* mutant plants.

| *APS1* | APS1-LP | 5´-GGTGGTCTTGTCTAGAGTGCAC-3´ |
| --- | --- | --- |
|  | APS1-RP | 5´-ACACACAGCCGCGTTATTTACCACCG-3´ |
|  | LBb1 T-DNA | 5´-GCGTGGACCGCTTGCTGCAACT-3´ |
| *pPGM* | pPGM-LP | 5´-TTGGGATTCTCCACTTTGTTG-3´ |
|  | pPGM-RP | 5´-AATACATACCGGTTTGGCTCC-3´ |
|  | GABI T-DNA | 5´-CCCATTTGGACGTGAATGTAGACAC-3´ |
